# Acute Upper Airway Obstruction Due to Neck Hematoma After Cervical Liposuction

**DOI:** 10.7759/cureus.102531

**Published:** 2026-01-29

**Authors:** Ahmed M Salloum, Alya Almadani, Palna Kulkarni, Ahmed Alghafri, Habiba Abouelenen

**Affiliations:** 1 Oral and Maxillofacial Surgery, Al Qassimi Hospital, Sharjah, ARE; 2 Dentistry, Thumbay Dental Hospital, Ajman, ARE

**Keywords:** angiogram, bleeding, double chin, hematoma, lipofilling, liposuction

## Abstract

Liposuction, also known as suction lipectomy, is an aesthetic procedure that removes fat from the submandibular, submental, chin, and neck regions. Hematomas are complications caused by ruptured blood vessels during the insertion of the cannula, which is more common in patients who use anticoagulant or anti-inflammatory medications before surgery. It could potentially be life-threatening if left untreated due to fatal airway obstruction and respiratory distress. Patients present with dysphagia, asphyxia, dysphonia, and a lot of discomfort. Immediate surgical intervention and pain control are necessary to provide patients with a positive quality of life. A 20-year-old male patient underwent liposuction under local anesthesia for treatment of his double chin. He presented one week later with slight swelling in the neck area, where vigorous manipulation and massaging had been done, and suddenly developed a massive, increasing swelling in the neck. Furthermore, the patient complained of difficulty breathing and swallowing. A diffuse hematoma was present in the subcutaneous plane, as identified by a computed tomography angiogram (CTA).

## Introduction

Double chin lipectomy or liposuction is done to remove adipose tissue between two layers, the skin and the platysma, at the subplatysmal level [1]. It has been seen in greater demand due to the enhanced aesthetic concerns of the population [1,2]. It is performed by inserting a cannula and aspirating through a 1-2 cm depth inside the subplatysmal layer [3]. One of the rare but serious complications of this procedure is hematoma formation [1,2]. It has been demonstrated that there is a lack of information in the literature about hematoma formation after liposuction cases. However, the risk of hematoma formation increases when volumes exceed 100 mL per unit of body mass index (BMI) [1]. It has also been observed that an elevation in blood pressure results in a 2.6 times higher incidence of hematoma. Moreover, if the patient is hypotensive, it can mask bleeding and result in delayed recognition [2]. Although airway-threatening cervical hematomas after submental liposuction are rare, they represent a recognized cause of delayed and potentially fatal airway obstruction [3]. Cross-sectional imaging, particularly computed tomography angiography (CTA), plays a key role in evaluating the extent of the hematoma, identifying active bleeding, and assessing supraglottic airway compromise. Adjunctive three-dimensional imaging modalities have also been used to objectively document airway narrowing and support vascular imaging in selected cases [4,5].

Surgical planning should consider the anatomical characteristics of the submental region and use techniques that minimize trauma. The use of smaller caliber cannulas, knowing the correct depth, and careful manipulation of tissues help reduce the chances of complications. The surgeon must have experience and a deep understanding of facial anatomy to avoid damage to nerves and blood vessels [1,3]. A surgeon should aim to restore not only the aesthetics, but also the anatomy and function of the patient's head and neck. Formulating an effective treatment plan based on the correct diagnosis is crucial to provide patients with the necessary help [3].

Postoperative management also plays a crucial role in preventing complications. Strict guidelines, such as avoiding intense physical activities in the first weeks, can help reduce hematomas [1]. Additionally, regular follow-up enables the early identification of any complications, which is crucial for effective treatment. In terms of technical advances, laser-assisted liposuction has been used as an alternative that can reduce the risks of complications [6]. The laser technique promotes controlled heating of the tissues, aiding in skin retraction and reducing mechanical trauma. This approach can be beneficial in patients with less elastic skin, offering a more harmonious result with a lower likelihood of complications [6].

## Case presentation

A 20-year-old male patient presented to the emergency department complaining of progressive neck swelling, which was increasing in size. Upon examination, he was suffering from progressive neck swelling, severe pain, and difficulty in swallowing, and he started to show signs of oxygen desaturation. Vital monitoring revealed an oxygen saturation of 87% on room air, which improved with supplemental oxygen via nasal cannula and oral mask. Intraoral examination showed mild edema of the floor of the mouth.

The patient was urgently admitted to the emergency department, and an initial diagnosis was performed, revealing a history of a cervicofacial liposuction procedure to treat double chin deformity under local anesthesia, which was performed in a private clinic seven days before the initial presentation. One week after the procedure, the operating surgeon performed a manual massage of the submandibular and submental areas, which preceded the sudden worsening of the swelling. Moreover, laboratory investigations, including complete blood count and coagulation profile, were within normal limits. The patient was started on intravenous fluids, empirical antibiotics consisting of amoxicillin and clavulanic acid 1.2 g twice daily, analgesics with Paracetamol 1 g every six hours, and dexamethasone 12 mg three times daily. The patient had no known medical conditions.

**Figure 1:**
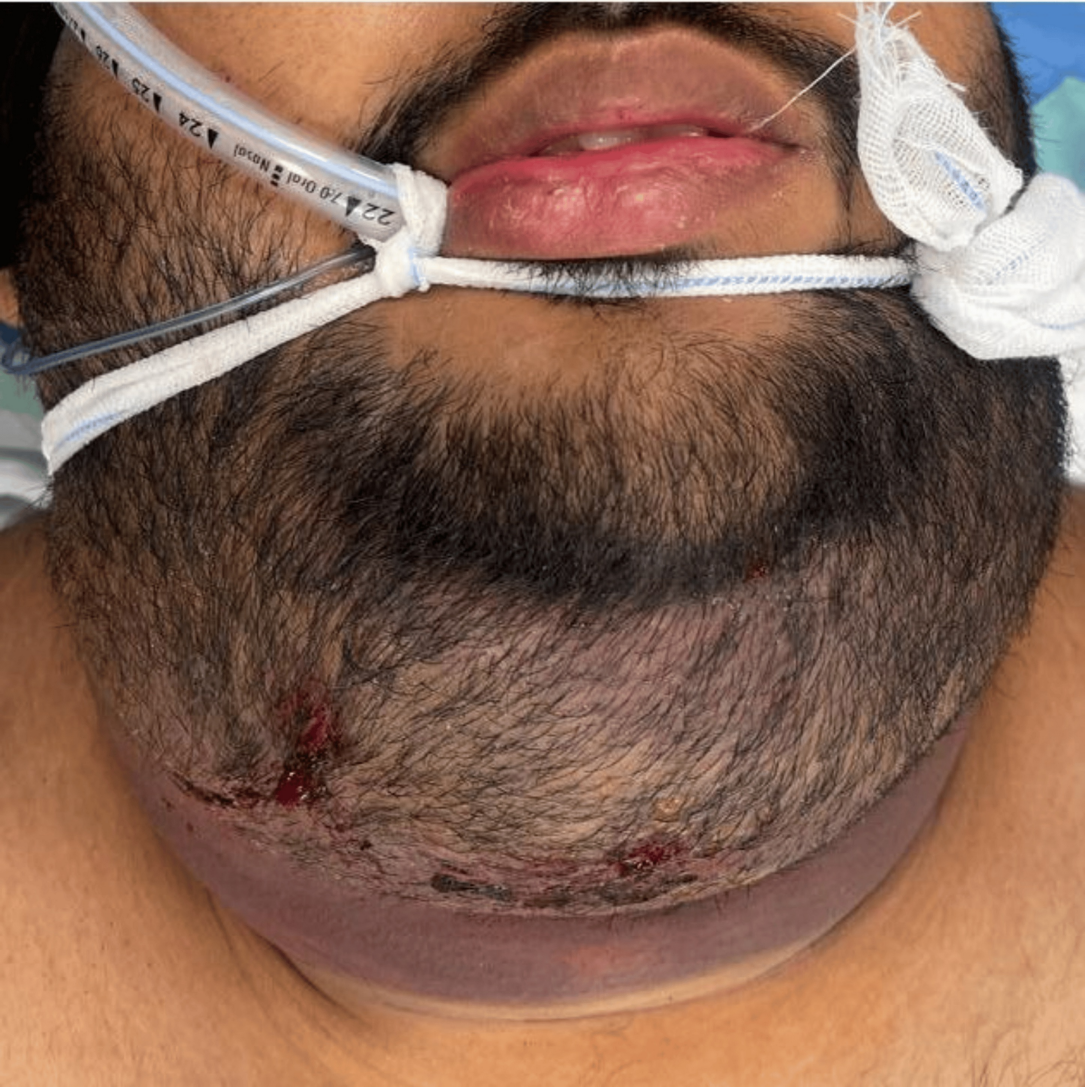
Submandibular swelling demonstrating a large hematoma with significant associated fluid collection.

After a detailed discussion with the patient and his family, it was revealed that the patient had a history of concerns regarding a double chin and was examined by a plastic surgeon, who advised liposuction to address the complaint. The surgery was performed under local anesthesia, and according to the patient's notes, it was completed without complications. Meanwhile, the patient presented again after seven days, and he developed an indurated submandibular swelling (Figure 1). The operating surgeon started to do a manual massage on the area of the submandibular and submental swelling, but it showed progressive swelling, pain, and dysphagia. 

**Figure 2:**
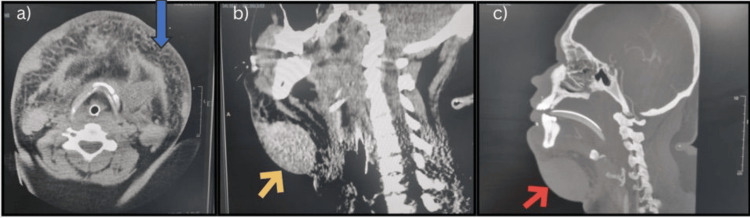
(a) Axial contrast CT showing subcutaneous high absorbance in the neck and bilateral submandibular area (blue arrow). (b) Sagittal contrast CT showing subcutaneous swelling and supraglottic narrowing (yellow arrow). (c) Sagittal contrast-enhanced CT showing extravasation (red arrow) in the lateral area of the right submandibular gland.

The patient was urgently admitted to the intensive care unit (ICU) to secure the airway and manage pain. Orotracheal intubation was done to ensure the airway, as well as supportive measures to control the swelling and pain. Management aimed to control the diagnosis of progressive swelling and control the general patient condition. CTA of the head and neck showed no injury to the major vessels, including the terminal head and neck vessels. It did, however, reveal disseminated subcutaneous hematoma collection (Figure 2).

**Figure 3:**
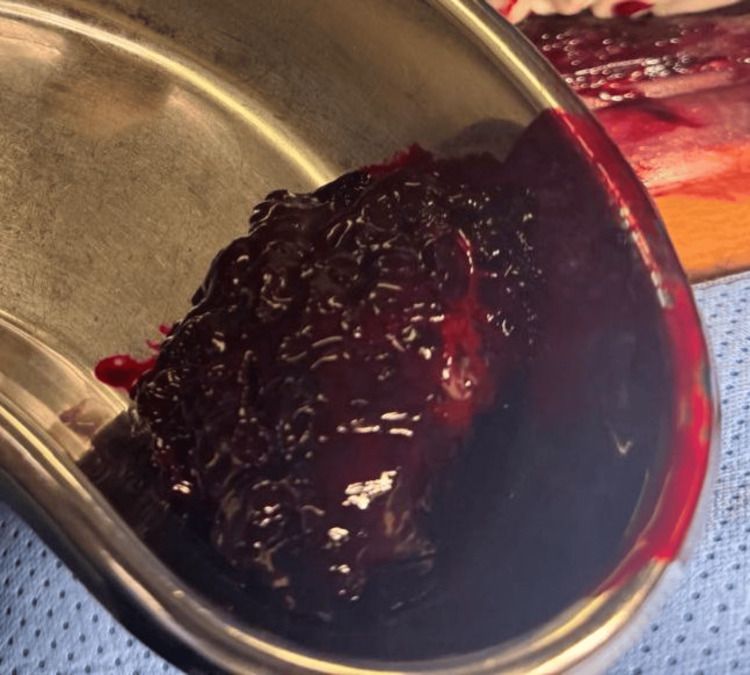
Evacuated hematoma measuring approximately 500 mL.

**Figure 4:**
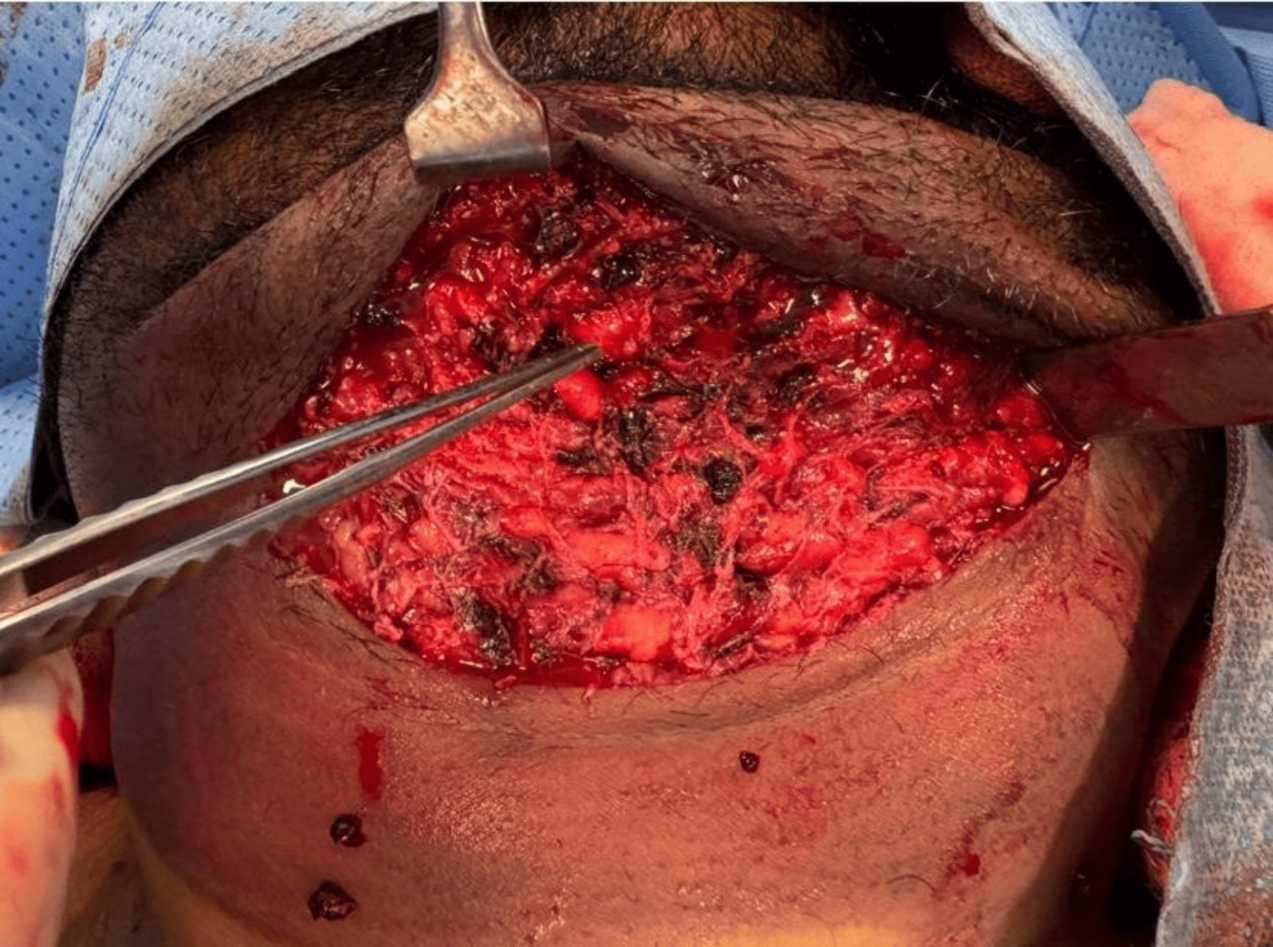
Internal appearance of the submandibular area showing subcutaneous bleeding and muscular contusion. The subcutaneous tissue is exfoliated, forming a pocket-like structure that contains a hematoma.

Twenty-four hours later, the swelling progressively increased as an indurated mass. The patient was then transported to the operating room, where consultation with the ear, nose, and throat (ENT) specialist was done, and he was ready for a tracheostomy. Under general anesthesia, a neck incision was performed to evacuate the massive hematoma (Figure 3). Following that, local exploration was performed to identify and control the bleeding vessels and points, ensuring hemostasis of the disseminated bleeding (Figure 4). Hemostasis was achieved using a bipolar coagulation device and application of a fibrin sealant, TISSEEL Lyo (Baxter Medical Products GmbH, Austria). Closure was done in layers, and the patient was transferred safely to the ICU, where he showed a progressive decrease in the hematoma size. The patient was uneventfully extubated and discharged after 48 hours.

## Discussion

In some cases, liposuction can be associated with some intraoperative or preoperative complications. The main complications associated with submental liposuction include prolonged edema, hematomas, dyspnea, delayed respiratory complications, fatal airway obstruction, and, in rare cases, tissue necrosis [3]. Edema is a common and expected reaction, but it may be prolonged in patients prone to fluid retention. Among the contraindications for submental liposuction are health conditions that compromise healing or increase the risk of infections, such as uncontrolled diabetes, severe heart problems, and coagulation disorders. Patients with active infections in the submental region, as well as heavy smokers, are also generally advised against undergoing the procedure, as smoking compromises vascularization and can hinder recovery. Rigorous medical evaluations and a detailed clinical history are essential to minimize the risks associated with these conditions [1]. Although females have a higher chance of developing hematomas due to their high esthetic demands, being a male and having other procedures done with liposuction is a great risk factor, as seen in our case [1,3]. Furthermore, the patient must have skin with good elasticity, as skin retraction after fat removal is necessary for satisfactory aesthetic results. Individuals with excessively loose skin may not be the best candidates for this technique and may require a cervical lift or other procedures [1].

This case provides surgeons with a valuable learning opportunity and highlights the challenges and complications that come with liposuction, such as hematoma formation seen in our patient following his liposuction surgery. Hematomas may occur due to the rupture of small blood vessels during the insertion of the cannula, being more common in patients who use anticoagulant or anti-inflammatory medications before surgery [3]. Moreover, tissue necrosis is one of the most serious complications of submental liposuction. This problem occurs when there is a disruption in blood circulation in the treated area, leading to the death of local tissues. Necrosis can be triggered by excessive trauma during the procedure or by an adverse reaction to the tumescent solution and requires immediate intervention to prevent further damage. Treatment involves the removal of necrotic tissue, followed by specific care to promote healing [3].

The prevention of complications in submental liposuction begins with a detailed preoperative evaluation, aiming to identify risk factors and appropriately select patients. Our case report promotes proper surgical planning, as we should consider the anatomical characteristics of the submental region and use techniques that minimize trauma. The use of smaller caliber cannulas, as well as careful manipulation of tissues, helps reduce the chances of complications. The surgeon must have experience and a deep understanding of facial anatomy to avoid damage to nerves and blood vessels [3]. Such an example can be the facial artery. The facial artery is known to originate from the external carotid artery and travels deep to the stylohyoid and digastric muscles [3,7]. It then courses along the inferior border of the mandible, passes behind the submandibular gland, and emerges onto the anterior surface of the mandible [3,7]. However, the pathway of this artery can vary greatly between individuals, and no major vessels were affected due to constant monitoring and early admission of our patient. Recent studies have shown that frequent movements of the jaw and neck contribute to the facial artery’s highly branched and winding nature. Surgeons must recognize these anatomical variations, particularly during classical liposuction, which is commonly performed worldwide [3,7,8].

Dealing with airway obstruction is crucial to ensure patient safety, especially in the delayed onset of fatal airway obstruction [3]. Draining hematomas or surgically excising them improves and relieves patients' airways. As drainage was not possible due to the size and progression of the hematoma, surgical excision was performed. In the event of complete airway obstruction, it is essential to intubate the patient, as leaving them unattended is fatal [9]. We made sure of that using orotracheal intubation; however, patient anxiety has also been linked with increased respiratory distress and elevated blood pressure [1,9]. Preoperative blood pressure exceeding 150/100 mmHg has been linked to a 2.6-fold increase in the risk of developing a hematoma [1]. It is important to leave a thin layer of subcutaneous fat during liposuction to prevent visible adhesions and protect the subdermal plexus. Additionally, subplatysmal fat should not be aspirated due to the possibility of developing submental cervical depression [1,9].

Immediate treatment with broad-spectrum antibiotics is necessary to contain bacterial spread, due to the incubation period of bacteria being 1-8 weeks, and prevent serious complications, such as abscess formation [1]. In addition to that, more serious infections can continue to develop if the condition is left untreated, such as necrotizing facial fasciitis [1]. We performed the surgery under general anesthesia, as it is preferred that the surgery be done that way to manage and maintain the patient’s blood pressure and positioning of the patient's head [1,9]. Understanding the complications in double chin liposuction is an important practice for refining physicians’ practices and developing strategies for a safer execution of the procedure.

Double chin liposuction, although considered a relatively safe procedure, presents a series of possible complications that, if not managed effectively, as seen in our case, can significantly compromise the aesthetic result and the patient's health. Furthermore, the technique used and the physician's experience proved to be determinants for minimizing these risks, reinforcing the importance of adequate training and rigorous safety protocols. Additionally, studies could provide more robust evidence on the effectiveness of preventive techniques and interventions to reduce the risks associated with the procedure [1-3]. The creation of standardized guidelines and consensus among professionals can help homogenize practices, benefiting both patients and clinicians in the field of aesthetic surgery [1-3]. Working alongside other healthcare professionals, such as nurses, anesthesiologists, and radiologists, is important to ensure that the patient gets the most effective treatment [10]. Our case report encourages further research and innovations in this understudied field.

## Conclusions

In our case, we encountered a patient who developed severe cervical swelling following cervicofacial liposuction performed under local anesthesia to correct a double chin. Postoperative care and close patient monitoring are crucial and can be lifesaving in such situations. This case serves as a reminder for surgeons to be thoroughly familiar with the anatomy of the head and neck and to recognize the limitations of the procedure. Furthermore, it highlights the importance of acknowledging the surgeon’s own capabilities and respecting the learning curve required when planning and performing such surgeries.
